# *Pseudomonas aeruginosa* Isolates among Clinical Samples showing Growth in a Tertiary Care Centre: A Descriptive Cross-sectional Study

**DOI:** 10.31729/jnma.6517

**Published:** 2022-08-31

**Authors:** Nabina Maharjan

**Affiliations:** 1 Department of Microbiology, Lumbini Medical College and Teaching Hospital, Tansen, Palpa, Nepal

**Keywords:** *beta-lactamase*, *multidrug resistant*, *Pseudomonas aeruginosa*

## Abstract

**Introduction:**

Carbapenems resistance due to metallo-beta-lactamase production in *Pseudomonas aeruginosa* is a major concern which is increasing globally resulting in limited therapeutic choices. This study aimed to find out the prevalence of *Pseudomonas aeruginosa* isolates among clinical samples showing growth of a tertiary care centre.

**Methods:**

A descriptive cross-sectional study was conducted on various clinical samples which showed growth in the Department of Microbiology of a tertiary care centre between 1 September 2020 to 28 February 2021 after receiving ethical approval from the Institutional Review Committee (Reference number: 03-G/020). Convenience sampling was done. All timely received clinical specimens were inoculated and incubated at 37°C for 48 hours and identified by standard microbiological techniques. Point estimate and 95% Confidence Interval were calculated.

**Results:**

Among 1049 clinical samples showing growth, 68 (6.48%) (4.99-7.97, 95% Confidence Interval) *Pseudomonas aeruginosa* were isolated. Among them, 6 (8.82%) were found to be metallo-beta-lactamase positive.

**Conclusions:**

The prevalence of *Pseudomonas aeruginosa* was similar to the studies done in similar settings. As metallo-beta-lactamase production was detected among isolated species which can be spread very rapidly and may develop a problematic scenario in treatment procedures, regular surveillance along with judicious use of carbapenems should precede.

## INTRODUCTION

*Pseudomonas aeruginosa* can develop antibiotic resistance by various mechanisms, enzyme production is the major mechanism of acquired resistance.^1^ Antibiotic resistance due to metallo-beta-lactamase (MBL) enzyme production is a global threat to antimicrobial therapy due to its rapid spread, potent carbapenemase activity, and resistance to beta-lactamase inhibitor and ability to hydrolyze all beta-lactam antibiotics except aztreonam.^2,3^

MBL-producing *Pseudomonas aeruginosa* was first reported in Japan in 1991, since then there is a substantial increase in reporting worldwide.^4^ The early and proper detection of MBL-producing *Pseudomonas aeruginosa* is crucial for properly treating critically ill patients and for the rapid initiation of strict infection control procedures to prevent nosocomial spread and dissemination of resistance.

The objective of this study was to find out the prevalence of *Pseudomonas aeruginosa* isolates among clinical samples showing growth in a tertiary care centre.

## METHODS

A descriptive cross-sectional study was conducted on various clinical samples showing growth processed in the Department of Microbiology of Lumbini Medical College and Teaching Hospital between 1 September 2020 to 28 February 2021 after receiving ethical approval from the Institutional Review Committee (Reference number: 03-G/020). All properly collected, timely delivered clinical specimens received in the study period which showed growth on culture were included whereas improperly collected dry swabs and lately delivered clinical samples showing growth which were were excluded. Convenience sampling was done and the sample size was calculated using the formula:


$$\begin{array}{ll} \text{n} & ={\text{Z}}^{2}\times \frac{\text{p}\times \text{q}}{{\text{e}}^{\text{2}}}\\ & =1.96^{2}\times \frac{0.0415\times 0.9585}{0.02^{2}}\\ & =383 \end{array}$$

Where,

n= minimum required sample size
Z= 1.96 at 95% Confidence Interval (CI)
p= prevalence of *Pseudomonas aeruginosa* isolate, 4.15%^5^
q= 1-p
e= margin of error, 2%

The required minimum sample size calculated was 383. Doubling the sample size, we get 766 but a sample size of 1049 samples was taken for the study. Various clinical samples (urine, pus/wound swab, blood, high vaginal swab, sputum, endotracheal specimen and stone) were collected from both sexes and all aged patients from various departments. All timely received clinical specimens were inoculated on Mac Conkey agar, Blood agar, and Chocolate agar and were incubated at 37°C for 48 hours. Identification of the bacteria was done by standard microbiological techniques like by study of morphological characteristics of isolated colonies along with observing pigment production, staining reaction and various biochemical properties.^6^ All isolated *Pseudomonas aeruginosa* were subjected to in vitro antibiotic sensitivity test Kirby-Bauer disc diffusion method as recommended by Clinical Laboratory Standard Institute.^7^

All imipenem-resistant *Pseudomonas aeruginosa* were screened as possible MBL producers. So all screened positive isolates were tested for MBL production by Imipenem-EDTA combined disk method (CDT) and Double disk synergy test (DDST). In CDT two imipenem disks (10 |jg) were placed 25 mm apart from centre to centre. Then 10 microlitre of 0.5 M Ethylenediamine tetraacetic acid (EDTA) was added. An increase in inhibition zone diameter of ≥7 mm in the imipenem-EDTA disk as compared to the imipenem disk alone was considered as MBL producers.^8^ In DDST imipenem disc (10 μg) was placed on Mueller Hinton Agar media inoculated with test organism and 10 mm apart a blank filter paper disc was placed to which 0.5 M 10 microlitre EDTA solution was added. Enhancement of zone of inhibition between Imipenem and EDTA disc was considered MBL positive.^9^

Data were entered and analysed in IBM SPSS Statistics 18.0. Point estimate and 95% Confidence Interval were calculated.

## RESULTS

Among 1049 clinical samples showing growth, 68 (6.48%) (4.99-7.97, 95% CI) *Pseudomonas aeruginosa* were isolated. A total of 35 (51.47%) were isolated from male patients and 33 (48.52%) from female patients. Maximum *Pseudomonas aeruginosa* was isolated from the inpatient department 50 (73.52%) whereas 18 (26.47%) from the outpatient department. Total of 15 (22.05%) of *Pseudomonas aeruginosa* were isolated from the Department of Surgery, 14 (20.58%) from the Department of Obstetrics and Gynaecology, 13 (19.11%) from the Intensive Care Unit (ICU), 6 (8.82%) from the medical ward, 4 (5.88%) from Department of Emergency and 2 (2.94%) from Department of Orthopedics. The isolation of *Pseudomonas aeruginosa* in different age groups showed that 20 (29.41%), 18 (26.47%), 19 (27.94%) and 11 (16.17%) were from ≤20, 21-40, 41-60 and >60 age groups respectively. The mean age of patients with *Pseudomonas aeruginosa* infection was 37.13±23.94 years. The sample-wise distribution of *Pseudomonas aeruginosa* is tabulated below (Table 1).

**Table 1:** Sample-wise distribution of *Pseudomonas aeruginosa* (n= 68).

Sample	*Pseudomonas aeruginosa* n (%)
Pus	13 (19.11)
Urine	20 (29.41)
Sputum	7 (10.29)
Blood	8 (11.76)
High vaginal swabs	7 (10.29)
Endotracheal secretions	5 (7.35)
Stone	8 (11.76)

Out of 20 (29.41%) *Pseudomonas aeruginosa* isolated from urine sample, 3 (15%) were MBL producers. A total of 1 (14.28%) and 2 (40%) MBL producers are isolated from the sputum and endotracheal secretions respectively. Polymyxin B 63 (92.64%) followed by imipenem 61 (89.70%) were two most sensitive drug for *Pseudomonas aeruginosa* (Table 2).

**Table 2:** Antibiotic susceptibility pattern of *Pseudomonas aeruginosa* (n= 68).

Antibiotics	Sensitive n (%)	Intermediate n (%)	Resistant n (%)
Ciprofloxacin	44 (64.70)	5 (7.35)	19 (27.94)
Gentamicin	45 (66.17)	-	23 (33.82)
Amikacin	48 (70.58)	-	20 (29.41)
Ceftazidime	30 (44.11)	2 (2.94)	36 (52.94)
Piperacillin	38 (55.88)	6 (8.82)	24 (35.29)
Tobramycin	47 (69.11)	1 (1.47)	20 (29.41)
Polymyxin B	63 (92.64)	-	5 (7.35)
Azetronam	47 (69.11)	6 (8.82)	15 (22.05)
Piperacillin-Tazobactam	42 (61.76)	6 (8.82)	20 (29.41)
Imipemem	61 (89.70)	-	7 (10.29)

A total of 6 (8.82%) were found to be metallo-beta-lactamase-producing *Pseudomonas aeruginosa.* Out of which 5 (83.30%) MBL producing *Pseudomonas aeruginosa* were multidrug resistant (Table 3).

**Table 3:** Findings of MBL positive and MBL negative *Pseudomonas aeruginosa* (n= 68).

	MBL positive (n= 6) n (%)	MBL negative (n= 62) n (%)
**Antibiotic susceptibility**		
Ciprofloxacin	1(16.66)	43 (69.35)
Gentamicin	-	45 (72.58)
Amikacin	1 (16.66)	47 (75.80)
Ceftazidime	-	30 (48.38)
Piperacillin	1 (16.66)	37 (59.67)
Tobramycin	1 (16.66)	46 (74.19)
Polymyxin B	5 (83.33)	58 (93.50)
Azetronam	1 (16.66)	46 (74.19)
Imipenem	-	61 (98.38)
Piperacillin tazobactam	1 (16.66)	41 (66.12)
**Multidrug resistance**		
Multi drug-resistant (resistant to 3 or more antibiotic group)	5 (83.30)	16 (25.80)

## DISCUSSION

*Pseudomonas aeruginosa* is emerging as one of the leading cause of healthcare-associated infections worldwide. In our study, among 1049 samples showing growth of bacteria, *Pseudomonas aeruginosa* was isolated from 68 (6.48%). This prevalence rate was somewhat higher and lower than other studies 4.15% and 8.59% respectively.^5,10^ Among 68 total isolated *Pseudomonas aeruginosa* 53 (73.5%) were isolated from inpatient department patients and 18 (26.5%) from outpatient department patients. Similar to our study high rates of *Pseudomonas aeruginosa* were reported from the Inpatient departments in many other studies 83.1%, 73.22% and 80%.^3,2,11^

*Pseudomonas aeruginosa* causes serious infection mainly in immunocompromised hosts such as those with severe burn or wound surgery and also in those indwelling devices.^5,12^ In our study the highest number of *Pseudomonas aeruginosa* were isolated from urine samples followed by pus samples and least from endotracheal tubes. A similar observation was reported in another study.^11^ Most of the other studies showed the highest isolation from pus specimens.^5,13^ The high rate of isolation from the urine samples in our study may be due to its ability to cause urinary tract infections in most people.^12^

*Pseudomonas aeruginosa* can develop antibiotic resistance by various mechanisms, enzyme production is the major mechanism of acquired resistance.^1^ Among 10 different antibiotics that were tested to isolated bacteria, polymyxin B followed by imipenem were the two most effective drugs showing 92.6% and 89.7% sensitivity respectively. Ceftazidime was found to be the least effective drug showing 44.1% sensitivity. This result was found to be similar to results published by other studies.^14,15^

According to various studies MDR *Pseudomonas aeruginosa* ranges from 20 to 85%.^12^ In our study 21 (30.9%) of isolated *Pseudomonas aeruginosa* were multidrug-resistant that were resistant to more than 3 antibiotics belonging to different classes. Some studies showed higher MDR isolates than our study 69.1%, and 63.3%.^13,16^ Out of a total of 68 *Pseudomonas aeruginosa* isolated in our study 6 (8.8%) were found to be MBL positive. This was similar to the result shown in another study 8.7%.^13^ However higher rate of MBL-mediated resistance was reported by many different studies 18.2%, 21.8%, 22.4%, 30.9% and 31.4%.^3,14,17,19^ This variation in the prevalence of MBL in our study may be due to our geographical location, infection control practice and method of detection.^20^

In our study out of 7 imipenem-resistant *Pseudomonas aeruginosa*, 6 isolates were MBL positive by both CDT and DDST. But other studies showed a high number of MBL positivity by CDT compared to DDST.^16,17^ This may be due to the small sample size resulting in a low number of imipenem resistant isolates so that no more variation between the two test methods was found. Sixty percent of *Pseudomonas aeruginosa* isolated from endotracheal tube culture were positive for MBL production. There was another study which also reported maximum MBL positivity from endotracheal tip culture.^2^ Patients with indwelling devices act as the major risk factor for the development of resistance.^4^ All most all 83.3% of MBL producing *Pseudomonas aeruginosa* were MDR. All MBL-positive isolates showed almost completely resistant to ceftazidime, gentamicin and imipenem, whereas 16.7% of sensitivity was shown by ciprofloxacin, amikacin, tobramycin and aztreonam. Polymyxin B was sensitive to 83.3% of MBL-positive *Pseudomonas aeruginosa* so was remained the only effective drug of choice. Other studies also reported polymyxin B as the most effective antibiotic.^3,18^

In this study, phenotypic methods were used for the detection of MBL. The molecular method for detection of responsible gene sequence which is considered the gold standard was not done. Similarly, this study was done for a short period with small sample size. These were the limitations of this study.

## CONCLUSIONS

The prevalence of *Pseudomonas aeruginosa* in our hospital setting was found to be similar to other studies done in similar settings. Similarly, MBL producers among them were lower as compared with other different studies. As MBL-positive isolates exhibit resistance to many antibiotics belonging to different classes along one of the most effective antibiotic imipenem, it results in a very limited choice of antibiotic to treat, we should always monitor its prevalence and should always use carbapenems judiciously.
